# Malformation of a Lower Incisor

**Published:** 1849-07

**Authors:** 


*Malformation of a Lower Incisor.*—A little girl, about nine years of age, was brought to our office a few days since by her mother, for the purpose of having a painful temporary molaris extracted. After having performed the operation, our attention was directed to the left central lower incisor, which presented a very singular and anomalous appearance. Immediately above the margin of the gum, on the anterior surface, was a bulge or tubercle, extending about half way to the cutting edge of the tooth, and projecting about the twelfth of an inch, giving to the tooth somewhat the appearance of two teeth united. At the upper edge of the tubercle a very narrow superficial groove passes horizontally across the tooth. Above this, the appearance of the tooth is perfectly natural.—*Balt. Ed.*

